# Basics, Epidemiology, Diagnosis, and Management of Liver Tumor

**DOI:** 10.3390/jcm12020524

**Published:** 2023-01-09

**Authors:** Hiroyuki Abe, Kenya Kamimura

**Affiliations:** 1 Division of Gastroenterology and Hepatology, Graduate School of Medical and Dental Sciences, Niigata University, 1-757 Asahimachido-ri, Chuo-ku, Niigata 9518510, Japan; 2 Department of General Medicine, Niigata University School of Medicine, 1-757 Asahimachido-ri, Chuo-ku, Niigata 9518510, Japan

Liver tumors include both benign (non-cancerous) and malignant (cancerous) varieties [1], and the latter tumors are further classified into tumors that originated in the liver (primary liver cancer) and tumors that spread from extrahepatic primary tumors (metastatic liver cancer). Hepatocellular carcinoma (HCC) and cholangiocarcinoma are the two major malignant primary liver tumors [2], whereas colorectal, lung, and pancreatic cancers are the major extrahepatic primary tumors causing liver metastasis. A wide variety of therapeutic options can be applied to treat these tumors [3,4]; however, hepatic functional reserve is a limitation factor for the selection. Therefore, a correct understanding of the tumors, use of the newest guidelines, and therapeutic intervention for background liver disease are all essential for physicians to make correct diagnoses and therapeutic decisions. This special issue was launched to address these needs, seeking to provide the most current knowledge and understanding of basic and clinical information for the diagnosis and therapeutic management of liver tumors.

Niwa et al. have summarized the effects of cyclin D1-binding protein (CCNDBP1), which is considered to be a tumor suppressor, on HCC cell proliferation. They reported that CCNDBP1 activates ataxia telangiectasia mutated (ATM)—checkpoint kinase 2 (CHK2) pathway through the inhibition of EZH2 expression, assessing the gene expression in *CCNDBP1*-overexpressed cells and *Ccndbp1* knockout mice. As a result, the overexpression of *CCNDBP1* in HCC cells stimulated cell growth, showing resistance to chemotherapy and X-ray-induced DNA damage (“Cyclin D1 Binding Protein 1 Responds to DNA Damage through the ATM–CHK2 Pathway” by Niwa et al. [5]).

Horigome et al. further clarified the involvement of the *CCNDBP1*-*ATM*-*CHK2* pathway in the DNA damage response using dextran-sodium-sulfate (DSS)-induced colitis mice model. Interestingly, *Ccndbp1* knockout mice ameliorated DSS-induced colitis, an effect indicated by the histological score increase, disease activity index elevation, and colon length shortening. These results are related to the distal colon-dominant Atm and Chk2 phosphorylation, an increase in TdT-mediated dUTP-biotin nick end labeling, and cleaved caspase 3-positive cells in the colon. These results suggested that *Ccndbp1* contributed to activating the Atm–Chk2 pathway, in turn triggering inflammation and apoptosis of mucosal cells in the colon. (“Involvement of DNA Damage Response via the Ccndbp1-Atm-Chk2 Pathway in Mice with Dextran-Sodium-Sulfate-Induced Colitis” by Horirome et al. [6]). Based on this progress, it is essential to evaluate *CCNDBP1* expression in the human liver samples of various chronic liver diseases that cause inflammation and fibrotic changes, and ultimately to apply these results to novel molecular therapy for the liver tumor.

Surgical treatment for malignant liver tumor is an important therapeutic option for curability; however, the indication is based on the hepatic functional reserve. To improve function, portal vein embolization (PVE) has been performed to induce hepatic hypertrophy and increase hepatic functional capacity. Interestingly, Heise et al. reported the usefulness of artificial neural network (ANN) methods, using preoperative computed tomography images to predict maximum hepatic functional capacity and the possibility of hypertrophy following PVE (“CT-Based Prediction of Liver Function and Post-PVE Hypertrophy Using an Artificial Neural Network” by Heise et al. [7]). The results presented provide in-depth descriptions of next generation technology. These advances could further optimize clinical techniques in order to perform safe surgical liver tumor removal. 

Surgical resection is also a curative therapeutic option for the metastatic liver tumors of colorectal cancers. While various modifications had previously been made into surgical strategies, Krawczyk et al. focused on the Pringle maneuver (PM), which is used to prevent intraoperative bleeding during the performance of hepatectomy but conversely, it also causes temporal hepatic ischemia, potentially inducing a ischemia-reperfusion liver injury followed by the post-hepatectomy liver failure and generally increasing the postoperative risk of cancer recurrence. Krawczyk et al. have shown, in a clinical retrospective study, that PM use had no negative effects on long-term outcomes for patients undergoing major, oncologically radical liver resections for colorectal metastases. (“Long-Term Effects of Pedicle Clamping during Major Hepatectomy for Colorectal Liver Metastases” by Krawczyk et al. [8])

Liver tumors also include rare neoplasms, a category which encompasses perivascular epithelioid cell tumor (PEComa) and which is usually incidentally diagnosed. Therefore, the accumulation of knowledge and experience in diagnosing and treating cases is vitally important. PEComa is a rare malignant tumor derived from perivascular cells [9]. Krawczyk et al. reviewed the clinicopathological features of 20 PEComa cases, diagnosed in their institute in order to provide management guidance. They reported that tumors with high arterial vascularization are observed based on the radiological studies and that hepatic resection is the treatment of choice. (“PEComa—A Rare Liver Tumor” by Krawczyk et al. [10]).

These interesting studies and articles will enhance the ability of physicians to make the correct diagnosis and therapeutic decisions for liver tumors based on the knowledge and understanding of basic and clinical information alike. In addition, this Special Issue will seek to provide knowledge that will contribute to establishing new research plans to develop novel diagnostic markers, methodologies, and therapies.
